# A Novel INS and Doppler Sensors Calibration Method for Long Range Underwater Vehicle Navigation

**DOI:** 10.3390/s131114583

**Published:** 2013-10-28

**Authors:** Kanghua Tang, Jinling Wang, Wanli Li, Wenqi Wu

**Affiliations:** 1 College of Mechatronics and Automation, National University of Defense Technology, Changsha 410073, China; E-Mails: liwanlli1201@hotmail.com (W.L.); wenqiwu_lit@hotmail.com (W.W.); 2 School of Civil and Environmental Engineering, University of New South Wales, Sydney, NSW 2052, Australia; E-Mail: jinling.wang@unsw.edu.au

**Keywords:** inertial navigation system, Doppler velocity log, integrated navigation, autonomous underwater vehicle, parameters calibration, iterative implementation

## Abstract

Since the drifts of Inertial Navigation System (INS) solutions are inevitable and also grow over time, a Doppler Velocity Log (DVL) is used to aid the INS to restrain its error growth. Therefore, INS/DVL integration is a common approach for Autonomous Underwater Vehicle (AUV) navigation. The parameters including the scale factor of DVL and misalignments between INS and DVL are key factors which limit the accuracy of the INS/DVL integration. In this paper, a novel parameter calibration method is proposed. An iterative implementation of the method is designed to reduce the error caused by INS initial alignment. Furthermore, a simplified INS/DVL integration scheme is employed. The proposed method is evaluated with both river trial and sea trial data sets. Using 0.03°/h(1σ) ring laser gyroscopes, 5 × 10^−5^ g(1σ) quartz accelerometers and DVL with accuracy 0.5% V ± 0.5 cm/s, INS/DVL integrated navigation can reach an accuracy of about 1‰ of distance travelled (CEP) in a river trial and 2‰ of distance travelled (CEP) in a sea trial.

## 1. Introduction

Autonomous Underwater Vehicles (AUV) present a uniquely challenging navigational problem because they operate autonomously in a highly unstructured environment [1]. Autonomous operations in deep water or covert military operations require the AUV to handle submerged operation for long periods of time. Currently, few techniques exist for reliable navigation for long range AUVs. Ultra-short baseline (USBL) acoustic navigation systems are employed on industrial, military, and scientific underwater vehicles and are preferred for the task of docking a vehicle to a transponder-equipped docking station [2–6]. Terrain- or landmark-based navigation methods use real-time sensing and a terrain or landmark map (e.g., topographic, magnetic, gravitational, or other geodetic data) to determine the vehicle's position [2]. But an *a priori* map is seldom available in AUV terrain- or landmark-based navigation. The standard method for full ocean depth XYZ acoustic navigation is 12-kHz-long baseline (LBL) acoustic navigation [2,7], but the precision and update rate of LBL position fixes vary over several orders of magnitude depending on the acoustic frequency, range, and acoustic path geometry [2]. Global Navigation Satellite System (GNSS) provides superior three-dimensional navigation capability for both surface and air vehicles but its signal cannot be directly received by deeply submerged ocean vehicles. A strapdown inertial navigation system (INS) is a good choice for self-contained localization and navigation of AUVs, but its position error accumulates with time elapse due to the inherent bias errors of gyros and accelerometers. Hence a navigation system based on INS will have an unacceptable position error drift without sufficient aiding. INS/DVL integrated navigation system using the high accurate velocity offered by DVL to restrain the error accumulation of INS is a widely-used under-water integrated navigation technology [8–18]. Even when a DVL is included, the accuracy of INS/DVL integration will be reduced because of the scale factor error of DVL and the misalignments between INS and DVL.

Because the scale factor error of DVL and the misalignments between INS and DVL are the key factors which limit the accuracy of INS/DVL integration, calibration and compensation of these parameters must be done before a mission is conducted. This calibration is necessary to account for mechanical misalignments in the installations of the INS and DVL, as well as for potential errors in the velocity estimates of the units [7]. In practical engineering applications, the first adapted method is based on the assumptions: (a) both INS and DVL are mounted onto the same rigid structure throughout a mission; (b) the lever arms and misalignments between these devices remain constant and small. However, such assumptions are not realistic in the real world. The second adapted method is to treat the misalignments between INS and DVL as unknown and then GNSS is used to estimate misalignment parameters in three dimensions. However, only yaw misalignment parameter between INS and DVL was considered in some early work. For example, in [19], Joyce proposed a method to estimated yaw misalignment error by using least squares (LS) method. In [20,21], the heading accuracy was further considered as one of the key factors which limit the calibration accuracy. In [2,22,23], James and his colleagues improved the calibration method, with precise position of acoustic navigation sensors such as LBL, three dimensional misalignments between INS and DVL can then be estimated simultaneously. But this method is difficult to implement that it might cause some inconvenience for real applications. In [24], an online estimation method of DVL misalignment angle in SINS/DVL was presented. However, it requires the AUV to be operated with complex maneuvers to enhance observability of the unknown states. The paper proposes a novel alignment calibration method with external GNSS signals. However, there is no need to receive the GNSS signals continuously which make it suitable for AUV platforms. Furthermore, a recursive implementation which can eliminate the effects of the INS initial alignment is proposed. The accuracy of the calibration is further improved.

This paper is organized as follows: Section 2 introduces the navigation equations, including INS/DVL system equations and observation equations. The parameter calibration method is proposed in Section 3, followed by an iterative implementation to reduce the effects of the INS initial alignment. After the scale factor of DVL and misalignments between INS and DVL are fixed, the simplified INS/DVL integrated navigation system is designed in Section 4. An experimental evaluation of the proposed navigation system is presented in Section 5, where in particular, the performance of the navigation system both in the river trial and the sea trial is discussed. Finally, conclusions are drawn in Section 6.

## 2. Navigation Equations Model

**Figure 1.**
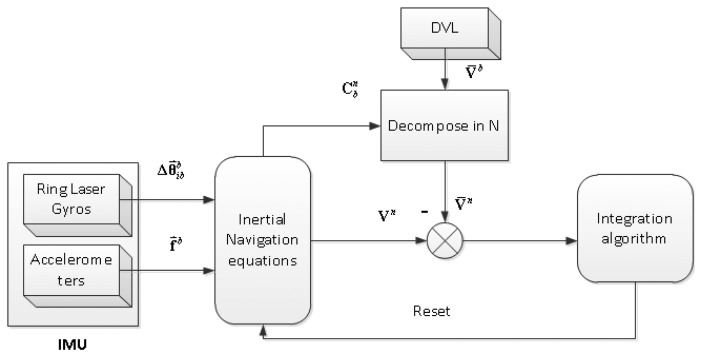
SINS/DVL integrated navigation.

The Autonomous Underwater Vehicle discussed in the paper is equipped with a DVL and an INS consisting of three gyroscopes and three accelerometers. The INS calculates position, velocity and attitude using high frequency data from an Inertial Measurement Unit. Navigation propagation equations are introduced in this section. The estimated states include position, velocity, attitude, biases of the inertial sensors, and biases of DVL. The typical integrated navigation scheme for AUV is shown in Figure 1. INS/DVL integration is employed for autonomous navigation for most of the time in the missions. Once the GNSS signals are available, the current position can be reset.

### 2.1. INS/DVL System Equations

The local level frame North-Up-East (NUE) is chosen as the navigation frame *n. b* is the INS body frame; *i* is the Earth-centered inertial (ECI) orthogonal reference frame; *e* denotes the Earth-centered Earth-fixed (ECEF) orthogonal reference frame. The states of the system model include position **P***^n^*, velocity **V***^n^*, attitude parameters through the direction cosine matrix $C_{b}^{n}$, gyro bias, **Δ**;*_g_* accelerometer bias **Δ***_a_* and errors in DVL. The DVL measurement error is mainly caused by the scale factor error *k* and the misalignment error *ε*, both of which can be regarded as an constant during the mission. The system equations can then be presented as [25,26]:

(1) $$\begin{array}{l} {\dot{\text{C}}}_{b}^{n}=C_{b}^{n}(\omega_{nb}^{b}\times ),\omega_{nb}^{b}=\omega_{ib}^{b}-\Delta_{g}-C_{n}^{b}(\omega_{ie}^{n}+\omega_{en}^{n})\\ {\dot{V}}_{e}^{n}=C_{b}^{n}(f^{b}-\Delta_{a})-(2\omega_{ie}^{n}+\omega_{en}^{n})\times V_{e}^{n}+g_{l}^{n}\\ {\dot{P}}^{n}=V^{n}\\ {\dot{\Delta }}_{g}=0\\ {\dot{\Delta }}_{a}=0 \end{array}$$

where $\omega_{nb}^{b}$ is the angular rate of the navigation frame relative to the body frame; $\omega_{ib}^{b}$ is the angular rate of the inertial frame relative to the body frame; $\omega_{ie}^{n}=\left[\omega_{ie}\cos L,\omega_{ie}\sin L,0\right]$ is the Earth's rotation rate in the navigation frame; *L* is the geographic latitude; *ω_ie_* is the Earth's rotation rate; $\omega_{en}^{n}$ is the angular rate of the navigation frame to the earth frame; **f***^b^* is the accelerometer measurement; $g_{l}^{n}$ is the local level gravitational acceleration expressed in the *n*-frame.

The scale factor error and the misalignments are assumed not to have a known time variation. Thus:

(2) $$\begin{array}{c} \dot{\varepsilon }=0\\ \dot{k}=0 \end{array}$$

### 2.2. Observation Equations

The velocity measurements **Ṽ***^d^* from DVL in the Doppler instrument frame *d* can be expressed as follows:

(3) $${\tilde{V}}^{d}=V^{d}+\delta V^{d}+kV^{d}$$

where $V^{d}={\left[\nu_{x}^{d},\nu_{y}^{d},\nu_{z}^{d}\right]}^{T}$ is the true value of the velocity of DVL, *k* is the scale factor error, **δV***^d^* presents the Gaussian white noise.

Therefore, the observation equation can be expressed as:

(4) $$y=(1+k)C_{b}^{d}C_{n}^{b}V_{\text{INS}}^{n}$$

where $C_{d}^{b}$ is the misalignment matrix between INS and DVL. It is the skew matrix of the misalignments **ε**.

## 3. Parameter Calibration Algorithm

The main advantage of the online calibration method proposed in [24] is that no external sensors are required. However, it requires the AUV to operate complex maneuvers. Generally, AUVs travel in a straight path at a constant velocity. Although the scale factor error and the misalignments can be chosen as the Kalman filter states for the INS/DVL integrated navigation system and hence estimated on line, it should be noted from the observability analysis that not all of the states are observable under that sailing condition [25–29]. Therefore, a novel parameter calibration method is proposed.

### 3.1. Formulas of the Proposed Method

It can be guaranteed that the misalignments ***ε*** are reduced to a small value during manufacture. Since the velocity of DVL in the lateral direction $\nu_{y}^{d}$ and the up direction $\nu_{z}^{d}$ are miniature, ignore the influence of the roll error *ε_x_* of the misalignments. Therefore, only the scale factor error *k*, the yaw misalignment error *ε_y_*, the pitch misalignment error *ε_z_* are considered.

Applying Equation (4), the DVL measurements can be expressed as:

(5) $${\tilde{\text{V}}}^{d}=(1+k)C_{b}^{d}C_{n}^{b}V^{n}=(1+k)[\text{I}-\varepsilon \times ]C_{n}^{b}V^{n}\approx [\text{I}-\varepsilon \times ]C_{n}^{b}V^{n}+kC_{n}^{b}V^{n}$$

Rearranging gives:

(6) $$-C_{b}^{n}\left[\varepsilon \times \right]C_{n}^{b}V^{n}+kV^{n}=C_{b}^{n}{\tilde{V}}^{d}-V^{n}$$

Ignoring the influence of the small products, so:

(7) $$-C_{b}^{n}\left[\varepsilon \times \right]{\tilde{V}}^{d}+kV^{n}=C_{b}^{n}{\tilde{V}}^{d}-V^{n}$$

Rearranging Equation (7) gives:

(8) $$C_{b}^{n}[{\tilde{V}}^{d}\times ]\varepsilon +kV^{n}=C_{b}^{n}{\tilde{V}}^{d}-V^{n}$$

During the process of calibration voyage, the AUV travels in a straight path at a constant velocity. Therefore, the roll *γ* and the pitch *θ* remain small:

(9) $$C_{b}^{n}={\left[C_{b}^{n}\right]}^{T}\approx \left[\begin{array}{ccc} \cos\varphi & \theta & -\sin\varphi \\ -\theta & 1 & \gamma \\ \sin\varphi & -\gamma & \cos\varphi \end{array}\right]$$

where *φ* denotes the yaw angle.

Substituting Equation (9) into Equation (8) gives:

(10) $$\left[\begin{array}{ccc} \cos\varphi & \theta & -\sin\varphi \\ -\theta & 1 & \gamma \\ \sin\varphi & -\gamma & \cos\varphi \end{array}\right]\;\left[\begin{array}{ccc} 0 & -{\tilde{\nu }}_{z}^{d} & {\tilde{\nu }}_{y}^{d}\\ {\tilde{\nu }}_{z}^{d} & 0 & -{\tilde{\nu }}_{x}^{d}\\ -{\tilde{\nu }}_{y}^{d} & {\tilde{\nu }}_{x}^{d} & 0 \end{array}\right]\varepsilon +kV^{n}=C_{b}^{n}{\tilde{V}}^{d}-V^{n}$$

where $\nu_{x}^{d}$ is the velocity of DVL in the forward direction.

Ignoring the influence of ${\tilde{\nu }}_{y}^{d}$, ${\tilde{\nu }}_{z}^{d}$ and small products gives:

(11) $$\left[\begin{array}{ccc} {\tilde{\nu }}_{y}^{d}\sin\varphi & -{\tilde{\nu }}_{z}^{d}\cos\varphi -{\tilde{\nu }}_{x}^{d}\sin\varphi & {\tilde{\nu }}_{y}^{d}\cos\varphi -\theta {\tilde{\nu }}_{x}^{d}\\ {\tilde{\nu }}_{z}^{d} & \gamma {\tilde{\nu }}_{x}^{d} & -{\tilde{\nu }}_{x}^{d}\\ -{\tilde{\nu }}_{y}^{d}\cos\varphi & -{\tilde{\nu }}_{z}^{d}\sin\varphi +{\tilde{\nu }}_{x}^{d}\cos\varphi & {\tilde{\nu }}_{y}^{d}\sin\varphi +\gamma {\tilde{\nu }}_{x}^{d} \end{array}\right]\varepsilon +kV^{n}=C_{b}^{n}{\tilde{V}}^{d}-V^{n}$$

Rearranging gives:

(12) $$\left[\begin{array}{ccc} {\tilde{\nu }}_{y}^{d}\sin\varphi & -{\tilde{\nu }}_{z}^{d}\cos\varphi -{\tilde{\nu }}_{x}^{d}\sin\varphi & {\tilde{\nu }}_{y}^{d}\cos\varphi -\theta {\tilde{\nu }}_{x}^{d}\\ {\tilde{\nu }}_{z}^{d} & \gamma {\tilde{\nu }}_{x}^{d} & -{\tilde{\nu }}_{x}^{d}\\ -{\tilde{\nu }}_{y}^{d}\cos\varphi & -{\tilde{\nu }}_{z}^{d}\sin\varphi +{\tilde{\nu }}_{x}^{d}\cos\varphi & {\tilde{\nu }}_{y}^{d}\sin\varphi +\gamma {\tilde{\nu }}_{x}^{d} \end{array}\right]\;\left[\begin{array}{c} \varepsilon_{x}\\ \varepsilon_{y}\\ \varepsilon_{z} \end{array}\right]+k\left[\begin{array}{c} \nu_{N}\\ 0\\ \nu_{E} \end{array}\right]=C_{b}^{n}{\tilde{V}}^{d}-\left[\begin{array}{c} \nu_{N}\\ 0\\ \nu_{E} \end{array}\right]$$

The misalignments **ε** can be regarded as small values, so:

(13) $$\left[\begin{array}{ccc} 0 & -{\tilde{\nu }}_{x}^{d}\sin\varphi & 0\\ 0 & 0 & -{\tilde{\nu }}_{x}^{d}\\ 0 & {\tilde{\nu }}_{x}^{d}\cos\varphi & 0 \end{array}\right]\;\left[\begin{array}{c} \varepsilon_{x}\\ \varepsilon_{y}\\ \varepsilon_{z} \end{array}\right]+k\left[\begin{array}{c} \nu_{N}\\ 0\\ \nu_{E} \end{array}\right]=C_{b}^{n}{\tilde{V}}^{d}-\left[\begin{array}{c} \nu_{N}\\ 0\\ \nu_{E} \end{array}\right]$$

From Equation (13), the following equation can be obtained:

(14) $$-{\tilde{\nu }}_{x}^{d}\varepsilon_{z}=\left[\begin{array}{ccc} 0 & 1 & 0 \end{array}\right]C_{b}^{n}{\tilde{V}}^{d}$$

Both parts of the Equation (14) are integrated to yield:

(15) $$\varepsilon_{z}=-\frac{\left[\begin{array}{ccc} 0 & 1 & 0 \end{array}\right]\int_{t_{0}}^{t_{1}}C_{b}^{n}{\tilde{V}}^{d}dt}{\int_{t_{0}}^{t_{1}}{\tilde{\nu }}_{x}^{d}dt}$$

where $\int_{t_{0}}^{t_{1}}{\tilde{\nu }}_{x}^{d}dt$ is the distance of the AUV travel during the time interval [*t*_0_,*t*_1_] it can be obtained by INS/DVL integrated navigation system. $\left[\begin{array}{ccc} 0 & 1 & 0 \end{array}\right]\int_{t_{0}}^{t_{1}}C_{b}^{n}{\tilde{V}}^{d}dt$ is the distance of the AUV travel in the up direction which can also be obtained by INS/DVL integrated navigation system.

From Equation (13):

(16) $$\left[\begin{array}{c} -{\tilde{\nu }}_{x}^{d}\sin\varphi \\ {\tilde{\nu }}_{x}^{d}\cos\varphi \end{array}\right]\;\varepsilon_{y}+k\left[\begin{array}{c} \nu_{N}\\ \nu_{E} \end{array}\right]=\left[\begin{array}{ccc} 1 & 0 & 0\\ 0 & 0 & 1 \end{array}\right]C_{b}^{n}{\tilde{V}}^{d}-\left[\begin{array}{c} \nu_{N}\\ \nu_{E} \end{array}\right]$$

Both parts are integrated to yield:

(17) $$\left[\begin{array}{c} -\int_{t_{0}}^{t_{1}}{\tilde{\nu }}_{x}^{d}\sin\varphi dt\\ \int_{t_{0}}^{t_{1}}{\tilde{\nu }}_{x}^{d}\cos\varphi dt \end{array}\right]\;\varepsilon_{y}+k\;\left[\begin{array}{c} \int_{t_{0}}^{t_{1}}\nu_{N}dt\\ \int_{t_{0}}^{t_{1}}\nu_{E}dt \end{array}\right]=\left[\begin{array}{ccc} 1 & 0 & 0\\ 0 & 0 & 1 \end{array}\right]\int_{t_{0}}^{t_{1}}C_{b}^{n}{\tilde{V}}^{d}dt-\left[\begin{array}{c} \int_{t_{0}}^{t_{1}}\nu_{N}dt\\ \int_{t_{0}}^{t_{1}}\nu_{E}dt \end{array}\right]$$

Dot-multiplying both of its parts by $\left[\begin{array}{c} \int_{t_{0}}^{t_{1}}-\nu_{E}dt\\ \int_{t_{0}}^{t_{1}}\nu_{N}dt \end{array}\right]$ gives:

(18) $${\left[\begin{array}{c} \int_{t_{0}}^{t_{1}}-\nu_{E}dt\\ \int_{t_{0}}^{t_{1}}\nu_{N}dt \end{array}\right]}^{T}\;\left[\begin{array}{c} -\int_{t_{0}}^{t_{1}}{\tilde{\nu }}_{x}^{d}\sin\varphi dt\\ \int_{t_{0}}^{t_{1}}{\tilde{\nu }}_{x}^{d}\cos\varphi dt \end{array}\right]\varepsilon_{y}+k{\left[\begin{array}{c} \int_{t_{0}}^{t_{1}}-\nu_{E}dt\\ \int_{t_{0}}^{t_{1}}\nu_{N}dt \end{array}\right]}^{T}\;\left[\begin{array}{c} \int_{t_{0}}^{t_{1}}\nu_{N}dt\\ \int_{t_{0}}^{t_{1}}\nu_{E}dt \end{array}\right]={\left[\begin{array}{c} \int_{t_{0}}^{t_{1}}-\nu_{E}dt\\ \int_{t_{0}}^{t_{1}}\nu_{N}dt \end{array}\right]}^{T}\;\left[\begin{array}{ccc} 1 & 0 & 0\\ 0 & 0 & 1 \end{array}\right]\;\int_{t_{0}}^{t_{1}}C_{b}^{n}{\tilde{V}}^{d}dt-{\left[\begin{array}{c} \int_{t_{0}}^{t_{1}}-\nu_{E}dt\\ \int_{t_{0}}^{t_{1}}\nu_{N}dt \end{array}\right]}^{T}\;\left[\begin{array}{c} \int_{t_{0}}^{t_{1}}\nu_{N}dt\\ \int_{t_{0}}^{t_{1}}\nu_{E}dt \end{array}\right]$$

Since:

(19) $${\left[\begin{array}{c} \int_{t_{0}}^{t_{1}}-\nu_{E}dt\\ \int_{t_{0}}^{t_{1}}\nu_{N}dt \end{array}\right]}^{T}\;\left[\begin{array}{c} \int_{t_{0}}^{t_{1}}\nu_{N}dt\\ \int_{t_{0}}^{t_{1}}\nu_{E}dt \end{array}\right]=0$$

Substituting this into Equation (18) gives:

(20) $$\varepsilon_{y}=\frac{{\left[\begin{array}{c} \int_{t_{0}}^{t_{1}}-\nu_{E}dt\\ \int_{t_{0}}^{t_{1}}\nu_{N}dt \end{array}\right]}^{T}\;\left[\begin{array}{ccc} 1 & 0 & 0\\ 0 & 0 & 1 \end{array}\right]\;\int_{t_{0}}^{t_{1}}C_{b}^{n}{\tilde{V}}^{d}dt}{{\left[\begin{array}{c} \int_{t_{0}}^{t_{1}}-\nu_{E}dt\\ \int_{t_{0}}^{t_{1}}\nu_{N}dt \end{array}\right]}^{T}\;\left[\begin{array}{c} -\int_{t_{0}}^{t_{1}}{\tilde{\nu }}_{x}^{d}\sin\varphi dt\\ \int_{t_{0}}^{t_{1}}{\tilde{\nu }}_{x}^{d}\cos\varphi dt \end{array}\right]}$$

Dot-multiplying both parts of Equation (17) by $\left[\begin{array}{c} \int_{t_{0}}^{t_{1}}{\tilde{\nu }}_{x}^{d}\cos\varphi dt\\ \int_{t_{0}}^{t_{1}}{\tilde{\nu }}_{x}^{d}\sin\varphi dt \end{array}\right]$ gives:

(21) $${\left[\begin{array}{c} \int_{t_{0}}^{t_{1}}{\tilde{\nu }}_{x}^{d}\cos\varphi dt\\ \int_{t_{0}}^{t_{1}}{\tilde{\nu }}_{x}^{d}\sin\varphi dt \end{array}\right]}^{T}\;\left[\begin{array}{c} -\int_{t_{0}}^{t_{1}}{\tilde{\nu }}_{x}^{d}\sin\varphi dt\\ \int_{t_{0}}^{t_{1}}{\tilde{\nu }}_{x}^{d}\cos\varphi dt \end{array}\right]\;\varepsilon_{y}+k{\left[\begin{array}{c} \int_{t_{0}}^{t_{1}}{\tilde{\nu }}_{x}^{d}\cos\varphi dt\\ \int_{t_{0}}^{t_{1}}{\tilde{\nu }}_{x}^{d}\sin\varphi dt \end{array}\right]}^{T}\;\left[\begin{array}{c} \int_{t_{0}}^{t_{1}}\nu_{N}dt\\ \int_{t_{0}}^{t_{1}}\nu_{E}dt \end{array}\right]={\left[\begin{array}{c} \int_{t_{0}}^{t_{1}}{\tilde{\nu }}_{x}^{d}\cos\varphi dt\\ \int_{t_{0}}^{t_{1}}{\tilde{\nu }}_{x}^{d}\sin\varphi dt \end{array}\right]}^{T}\;\left[\begin{array}{ccc} 1 & 0 & 0\\ 0 & 0 & 1 \end{array}\right]\;\int_{t_{0}}^{t_{1}}C_{b}^{n}{\tilde{V}}^{d}dt-{\left[\begin{array}{c} \int_{t_{0}}^{t_{1}}{\tilde{\nu }}_{x}^{d}\cos\varphi dt\\ \int_{t_{0}}^{t_{1}}{\tilde{\nu }}_{x}^{d}\sin\varphi dt \end{array}\right]}^{T}\;\left[\begin{array}{c} \int_{t_{0}}^{t_{1}}\nu_{N}dt\\ \int_{t_{0}}^{t_{1}}\nu_{E}dt \end{array}\right]$$

Since:

(22) $${\left[\begin{array}{c} \int_{t_{0}}^{t_{1}}{\tilde{\nu }}_{x}^{d}\cos\varphi dt\\ \int_{t_{0}}^{t_{1}}{\tilde{\nu }}_{x}^{d}\sin\varphi dt \end{array}\right]}^{T}\;\left[\begin{array}{c} -\int_{t_{0}}^{t_{1}}{\tilde{\nu }}_{x}^{d}\sin\varphi dt\\ \int_{t_{0}}^{t_{1}}{\tilde{\nu }}_{x}^{d}\cos\varphi dt \end{array}\right]=0$$

Substituting this into Equation (21), the scale factor can be calculated as follows:

(23) $$k+1=\frac{{\left[\begin{array}{c} \int_{t_{0}}^{t_{1}}{\tilde{\nu }}_{x}^{d}\cos\varphi dt\\ \int_{t_{0}}^{t_{1}}{\tilde{\nu }}_{x}^{d}\sin\varphi dt \end{array}\right]}^{T}\;\left[\begin{array}{ccc} 1 & 0 & 0\\ 0 & 0 & 1 \end{array}\right]\;\int_{t_{0}}^{t_{1}}C_{b}^{n}{\tilde{V}}^{d}dt}{{\left[\begin{array}{c} \int_{t_{0}}^{t_{1}}{\tilde{\nu }}_{x}^{d}\cos\varphi dt\\ \int_{t_{0}}^{t_{1}}{\tilde{\nu }}_{x}^{d}\sin\varphi dt \end{array}\right]}^{T}\;\left[\begin{array}{c} \int_{t_{0}}^{t_{1}}\nu_{N}dt\\ \int_{t_{0}}^{t_{1}}\nu_{E}dt \end{array}\right]}$$

Supposing the AUV travels in a straight path at a constant speed during [*t*_0_, *t*_1_], then:

(24) $$\begin{array}{l} \int_{t_{0}}^{t_{1}}\nu_{N}dt=[L_{G}(t_{1})-L_{G}(t_{0})]R\\ \int_{t_{0}}^{t_{1}}\nu_{E}dt=[\lambda_{G}(t_{1})-\lambda_{G}(t_{0})]R\cos[(L_{G}(t_{1})+L_{G}(t_{0}))/2] \end{array}$$

(25) $$\left[\begin{array}{c} \int_{t_{0}}^{t_{1}}{\tilde{\nu }}_{x}^{d}\cos\varphi dt\\ \int_{t_{0}}^{t_{1}}{\tilde{\nu }}_{x}^{d}\sin\varphi dt \end{array}\right]\approx \left[\begin{array}{ccc} 1 & 0 & 0\\ 0 & 0 & 1 \end{array}\right]\int_{t_{0}}^{t_{1}}C_{b}^{n}{\tilde{V}}^{d}dt=\left[\begin{array}{c} {}[L_{D}(t_{1})-L_{D}(t_{0})]R\\ \left[\lambda_{D}(t_{1})-\lambda_{D}(t_{0})]R\cos[(L_{D}(t_{1})+L_{D}(t_{0}))/2\right] \end{array}\right]$$

(26) $$\int_{t_{0}}^{t_{1}}{\tilde{\nu }}_{x}^{d}dt=\sqrt{{\left([L_{D}(t_{1})-L_{D}(t_{0})]R\right)}^{2}+{\left(\left[\lambda_{D}(t_{1})-\lambda_{D}(t_{0})]R\cos[(L_{D}(t_{1})+L_{D}(t_{0}))/2\right]\right)}^{2}}$$

(27) $$\int_{t_{0}}^{t_{1}}{\tilde{\nu }}_{x}^{d}dt=[\begin{array}{ccc} 0 & 1 & 0 \end{array}]\int_{t_{0}}^{t_{1}}C_{b}^{n}{\tilde{V}}^{d}dt=h_{D}(t_{1})-h_{D}(t_{0})$$

where *L_G_*(*t*_0_) and *L_G_*(*t*_1_) are the geographic latitude obtained by GNSS at *t*_0_ and *t*_1_ respectively; *λ_G_*(*t*_0_) and *λ_G_*(*t*_1_) are the geographic longitude obtained by GNSS at *t*_0_ and *t*_1_ respectively; *L_D_*(*t*_0_) and *L_D_*(*t*_1_) are the geographic latitude obtained by INS/DVL integrated navigation system at *t*_0_ and *t*_1_ respectively; *λ_D_*(*t*_0_) and *λ_D_*(*t*_1_) are the geographic longitude obtained by INS/DVL integrated navigation system at *t*_0_ and *t*_1_ respectively; *R* is the radius of the Earth. That is to say, the scale factor error *k*, misalignment yaw error *ε_y_* and pitch misalignment error *ε_z_* can be obtained only with the positions of INS/DVL integrated navigation system and GNSS at *t*_0_ and *t*_1_ in the case that the AUV travels in a near straight path at a constant speed.

### 3.2. An Iterative Implementation

**Figure 2.**
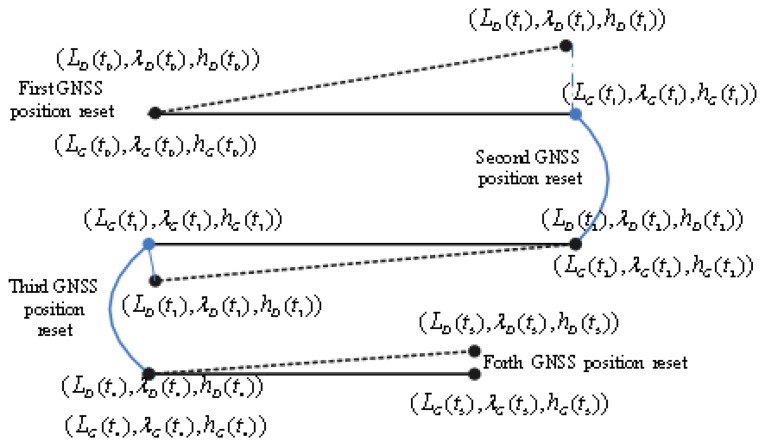
An iterative implementation scheme.

The attitude error caused by INS initial alignment is a key factor which limits the accuracy of the calibration. In [30,31], the methods of INS initial alignment for AUV are presented. In order to reduce the effects of the INS initial alignment, an iterative implementation is proposed as follows (shown in Figure 2):

(1) Update the position of INS/DVL integrated navigation by GNSS when the initial INS alignment is finished and record the positions (*L_D_*(*t*_0_),*λ_D_*(*t*_0_),*h_D_*(*t*_0_)) and (*L_G_*(*t*_0_),*λ_G_*(*t*_0_),*h_G_*(*t*_0_)).
(2) After the AUV has travelled over a distance, for example, 8 km, record the INS/DVL integrated navigation and GNSS positions as: (*L_D_*(*t*_1_),*λ_D_*(*t*_1_),*h_D_*(*t*_1_)) and (*L_G_*(*t*_1_),*λ_G_*(*t*_1_),*h_G_*(*t*_1_)).
(3) With the recorded position information from steps (1) and (2), the scale factor error *k*_0_, misalignment yaw error *ε_y0_* and pitch misalignment error *ε_z0_* can be obtained according to Equations (15), (20) and (23).
(4) The estimated scale factor and misalignment parameters are used in the subsequent navigation. Record the current position: (*L_D_*(*t*_2_),*λ_D_*(*t*_2_),*h_D_*(*t*_2_)), (*L_G_*(*t*_2_),*λ_G_*(*t*_2_),*h_G_*(*t*_2_)). Then the AUV takes a 180° turn. After the AUV has travelled over a distance, for example, 8 km, record more positions: (*L_D_*(*t*_3_),*λ_D_*(*t*_3_),*h_D_*(*t*_3_)) and (*L_G_*(*t*_3_),*λ_G_*(*t*_3_),*h_G_*(*t*_3_)).
(5) New parameter estimates (scale factor error *k*_1_, misalignment yaw error *ε_y1_* and pitch misalignment error *ε_z1_*) can be obtained by the newly recorded positions above. Therefore, the parameter estimates can be calculated as follows:
  (28) $$\begin{array}{ccc} 1+k=(1+k_{0})\times (1+k_{1}) & \varepsilon_{y}=\varepsilon_{y0}+\varepsilon_{y1} & \varepsilon_{z}=\varepsilon_{z0}+\varepsilon_{z1} \end{array}$$
(6) Repeat Step (4) and (5) until the accuracy of the INS/DVL integrated navigation system meets the requirement(about 1.5‰ of the distance travelled (CEP)).

## 4. Simplified INS/DVL Integrated Navigation System

Once the estimated parameters are fixed, state equations and observation equations of the INS/DVL integrated navigation system can be simplified.

### 4.1. Simplified INS/DVL Integrated Navigation System State Equations

After parameter calibration, the states of the Kalman filter can be reduced to: velocity error **δV**, attitude misalignments **ψ**, gyro bias **Δ***_g_*, accelerometer bias **Δ***_a_*. Thus, the INS partition of the state vector comprises the following 12 states:

(29) $$X(t)=[\delta V^{T},\psi^{T},\Delta_{g}^{T},\Delta_{a}^{T}]$$

The system state equation in a matrix form is given by:

(30) $$\dot{X}(t)=F(t)X(t)+W(t)$$

where **w**(***t***) denotes the Gaussian white noise. The system matrix F(t), expressed in term of 3 × 3 submatrices corresponding to the components of the state vector in Equation (29), is:

(31) $$F(t)=\left[\begin{array}{cccc} F_{11} & F_{12} & 0_{3\times 3} & F_{14}\\ F_{21} & F_{22} & F_{23} & 0_{3\times 3}\\ 0_{3\times 3} & 0_{3\times 3} & 0_{3\times 3} & 0_{3\times 3}\\ 0_{3\times 3} & 0_{3\times 3} & 0_{3\times 3} & 0_{3\times 3} \end{array}\right]$$

where:

(32) $$F_{11}=\left[\begin{array}{ccc} -\frac{V_{U}}{R} & -\frac{V_{N}}{R} & -2\omega_{U}-\frac{V_{E}\text{tgL}}{R}\\ \frac{2V_{N}}{R} & 0 & 2\omega_{N}+2\frac{V_{E}}{R}\\ 2\omega_{U}+\frac{V_{E}\text{tgL}}{R} & -2\omega_{N}-\frac{V_{E}\text{tgL}}{R} & \frac{V_{N}\text{tgL}}{R}-\frac{V_{U}}{R} \end{array}\right]$$

(33) $$F_{12}=\left[\begin{array}{ccc} 0 & -f_{E} & f_{U}\\ f_{E} & 0 & -f_{N}\\ -f_{U} & f_{N} & 0 \end{array}\right]$$

(34) $$F_{21}=\left[\begin{array}{ccc} 0 & 0 & \frac{1}{R}\\ 0 & 0 & \frac{\text{tgL}}{R}\\ -\frac{1}{R} & 0 & 0 \end{array}\right]$$

(35) $$F_{22}=\left[\begin{array}{ccc} 0 & -\frac{V_{N}}{R} & -\omega_{U}-\frac{V_{E}\text{tgL}}{R}\\ \frac{V_{N}}{R} & 0 & \omega_{N}+\frac{V_{E}}{R}\\ \omega_{U}+\frac{V_{E}\text{tgL}}{R} & -\omega_{N}-\frac{V_{E}}{R} & 0 \end{array}\right]$$

(36) $$F_{14}=C_{b}^{n}$$

(37) $$F_{23}=-C_{b}^{n}$$

where *ω_N_* = *ω_ie_* cos *L* and *ω_U_* = *ω_ie_* sin *L* are the Earth's rotation rates in the north and up directions, respectively.

### 4.2. Simplified INS/DVL System Observation Equations

The accuracy of the INS/DVL integrated navigation system is mainly caused by the inertial navigation attitude error, misalignments between INS and DVL and the velocity scale factor error of DVL, so:

(38) $${\hat{V}}_{\text{DVL}}^{n}={\tilde{C}}_{b}^{n}{\tilde{C}}_{d}^{b}V^{d}=[I_{3\times 3}-\psi \times ]C_{b}^{n}{\tilde{C}}_{d}^{b}V^{d}=C_{b}^{n}{\tilde{C}}_{d}^{b}V^{d}+C_{b}^{n}{\tilde{C}}_{d}^{b}V^{d}\times \psi =V^{n}+C_{b}^{n}{\tilde{C}}_{d}^{b}V^{d}\times \psi$$

Differencing the velocity of INS and DVL gives:

(39) $${\tilde{\text{V}}}_{\text{SINS}}^{n}-{\tilde{V}}_{\text{DVL}}^{n}=(V^{n}+{{\delta V}^{n}}_{\text{SINS}})-(V^{n}+{{\delta V}^{n}}_{\text{DVL}})={{\delta V}^{n}}_{\text{SINS}}-{{\delta V}^{n}}_{\text{DVL}}={{\delta V}^{n}}_{\text{SINS}}-C_{b}^{n}{\tilde{C}}_{d}^{b}V^{d}\times \psi$$

Once the scale factor and misalignments are determined by parameter calibration, the misalignment matrix ${\tilde{C}}_{d}^{b}$ between INS and DVL can be fixed. Based on velocity measurements **Ṽ***^d^* from DVL and the scale factor error, the true DVL velocity V^d^ is:

(40) $$V^{d}=\frac{{\tilde{V}}^{d}-\delta V}{1+k}$$

Combing Equations (39) and (40), the observation model is given by:

(41) $$Z={\tilde{V}}_{\text{SINS}}^{n}-{\tilde{V}}_{\text{DVL}}^{n}=HX(t)+V(t)$$

where:

(42) $$H=\left[\begin{array}{cccc} I_{3\times 3} & -C_{b}^{n}{\tilde{C}}_{b}^{n}V^{d}\times & 0_{3\times 3} & 0_{3\times 3} \end{array}\right]$$

and **v(t)** is the vector of the zero-mean Gaussian white noise.

Once the system state equations and observation equations are fixed, using the Kalman filter referred in [32,33], the states can be estimated.

## 5. Experimental Results and Discussions

Both river trials and sea trials were carried out to evaluate the performance of the proposed method. For this, an high performance INS Kit is designed. The INS Kit is a fully qualified inertial navigator that is based on three Ring Laser Gyros(RLG) produced by Huatian Photoelectron and INS Technology Co., Ltd., Changsha, China and three quartz accelerometers offered by Beijing StarNeto Technology Co., Ltd., Beijing, China. Inertial sensors specifications are shown Table 1.

The INS Kit modular architecture allows for various on board aiding devices such as GNSS, Doppler Velocity Log and Depth Sensor. The primary navigation aiding sensors are shown in Table 2.

**Figure 3.**
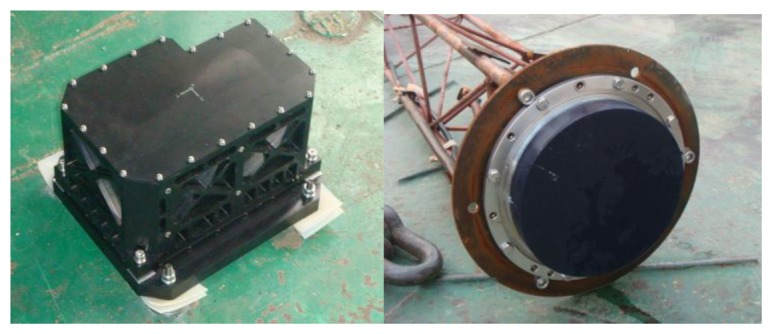
INS Kit and DVL modular.

The bottom-locked Doppler sensor HEU DVL produced by Harbin Engineering University could provide three-axis transformation velocities. The INS Kit and DVL modular are shown in Figure 3.

### 5.1. The River Trial

In the river trial, the devices were fixed on a ship. DGPS positioning results were employed as the benchmark. Four suiets of INS were fixed on the deck of a ship. They were marked with N1, N2, N3 and N4 respectively. The DVL modules were put 1 m underwater.

#### 5.1.1. River Trial Experimental Results

**Figure 4.**
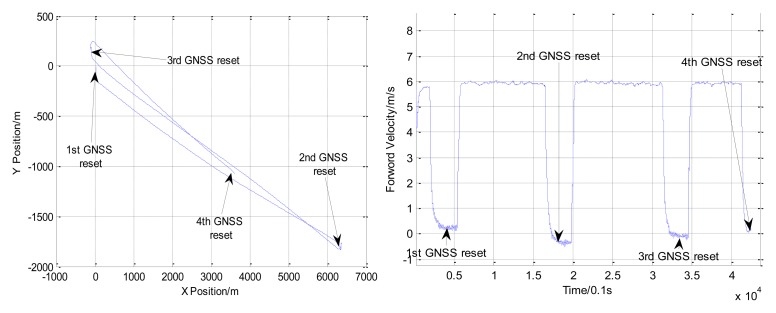
The trajectory and forward velocity of the vessel in the river trial.

A near straight trajectory which is about 8 km was chosen for parameter calibration. The trajectory and forward velocity of the vessel in the river trial are shown in Figure 4.

With the positions of the first and the second dot (shown in Figure 3) obtained from GNSS positions and INS/DVL positions, an initial scale factor of DVL and misalignments parameters were calculated. With the positions of the second and the third dot, the calibration parameters were updated with the proposed iterative implementation. These estimates are shown in Table 3.

With calibrated parameter estimates from Table 3 and the positions of the forth dot (shown in Figure 4), the final position errors are calculated in Table 4.

From Table 4, with calibrated parameter estimates, the final position errors of INS/DVL integrated navigation are within 5 m in the 7 km distance travelled. The accuracy of INS/DVL integrated navigation system is better than 1‰D (distance travelled). If the accuracy of INS/DVL integrated navigation system is bigger than 1.5‰D, with the positions of the third and the forth dot, the calibration parameters were updated with the iterative implementation.

#### 5.1.2. Validation of the Calibrated Parameters Estimates in the River Trial

**Figure 5.**
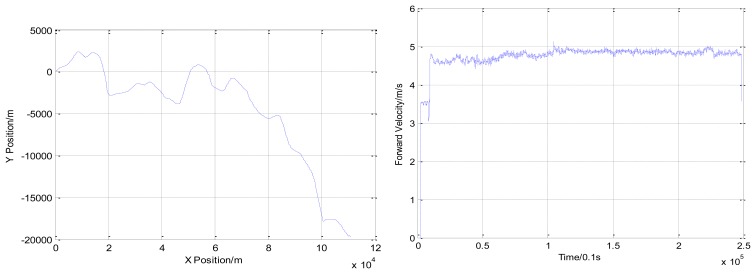
The trajectory and forward velocity of the vessel in a long distance river trial.

In order to evaluate the performance of the calibrated parameter estimates, another test was done. The calibrated parameter estimates were employed in the INS/DVL integrated navigation. During the experiment, the simplified INS/DVL integrated navigation scheme proposed in Section 4 is used. The trajectory of the vessel is shown in Figure 5. Comparing with the positions obtained from GNSS, the on-line experimental results are shown in Table 5.

As shown in Table 5, the accuracy of the INS/DVL integrated navigation system is about 1‰D(CEP). Furthermore, the accuracy of INS/DVL integrated navigation system is improved with the increase of distance travelled.

### 5.2. The Sea Trial

These experiments were done in the South Sea, China. During the sea trial, the INS and DVL were assembled as a single mechanical unit, and placed in the AUV. The scale factor and misalignments were calibrated in the river trial. In the sea trial, the experiment process of is as follows:

(1) INS initial alignment.
(2) The AUV is launched, and running an autonomous type of mission navigating with DVL aided INS and GNSS surface fixes at regular intervals. Once the DVL measurements are available, the navigation system is working at the mode of INS/DVL integrated navigation.
(3) After the AUV has travelled for a certain distance, it surfaces to receive the GNSS signals. By comparing the INS/DVL integrated navigation positioning data with independent DGNSS data, position error of the INS/DVL integrated navigation system can be obtained. Then the AUV submerges until the mission is finished.

Three experiments were carried out in the sea trial.

#### 5.2.1. Experiment 1

**Figure 6.**
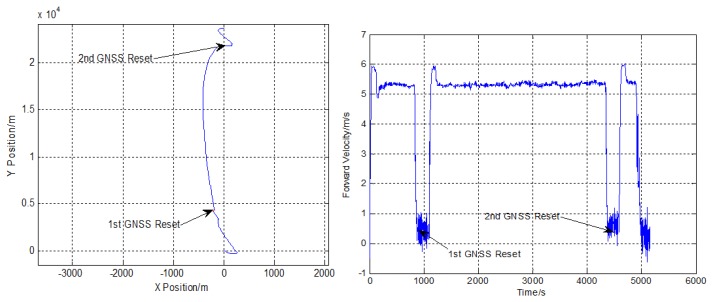
The trajectory and forward velocity of the AUV in Experiment 1.

In Experiment 1, after initial alignment, the AUV surfaced to receive DGPS measurements at the surface for a short period of time to calibrate the position error of INS/DVL integrated navigation system. Then the AUV submerged to travel a straight line approximately 17 Km in length, equivalent to about 2 h at nominal speed. The depth varies from 60 to 180 m. The trajectory obtained by INS/DVL integrated navigation system is shown in Figure 6. During the process of the experiment, the AUV surfaced twice to receive the GNSS signals.

Comparing with the positions obtained from GNSS, the accuracy of the INS/DVL integrated navigation system is shown in Table 6. The accuracy of the INS/DVL integration is about 1.76‰D.

#### 5.2.2. Experiment 2

**Figure 7.**
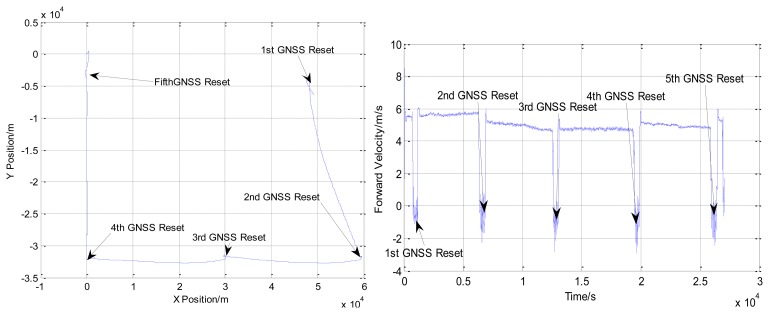
The trajectory and forward velocity of the AUV in Experiment 2.

In Experiment 2, the AUV travelled for about 120 km. The depth varies from 60 to 180 m. The trajectory obtained by INS/DVL integrated navigation system is shown in Figure 7. During the process of the experiment, the AUV surfaced five times to receive the GNSS signals.

Comparing the positions obtained from GNSS, the accuracy of the INS/DVL integrated navigation system is shown in Table 7. The accuracy of the INS/DVL integrated navigation system is within 3‰D.

#### 5.2.3. Experiment 3

**Figure 8.**
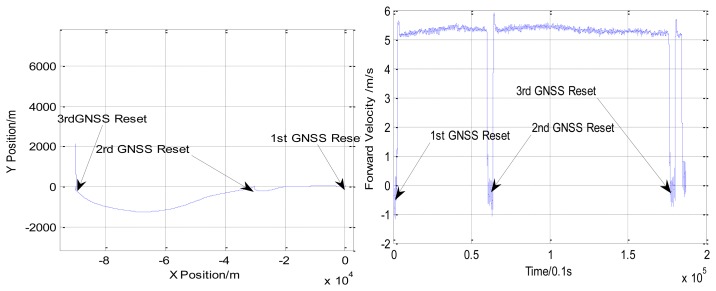
The trajectory and forward velocity of the AUV in Experiment 3.

In Experiment 3, the AUV travelled for about 90 km. The depth varies from 60 to 180 m. The trajectory obtained by INS/DVL integrated navigation system is shown in Figure 8. During the process of the experiment, the AUV surfaced three times to receive the GNSS signals.

Comparing the position obtained from DGNSS, the accuracy of the INS/DVL integrated navigation system is shown in Table 8. The accuracy of the INS/DVL integrated navigation system is within 3‰D.

According to the experimental results in Table 6, 7 and 8, the INS/DVL integrated navigation system has reached the accuracy of about 2‰D (CEP) with the calibrated parameter estimates obtained by the proposed method.

## 6. Conclusions

In order to meet the requirements of the AUV, an INS/DVL integrated navigation method has been designed. As the scale factor of DVL and misalignments between INS and DVL are the key factors which limit the accuracy of the INS/DVL integrated navigation, a novel parameter calibration method has been proposed. With this method, it is needless to receive GNSS signals continuously, making this method suitable for AUV platforms. The proposed method has been evaluated with both river trial and sea trial data sets. With the calibrated parameter estimates, INS/DVL integrated navigation can reach the accuracy of about 1‰ of the distance travelled (CEP) in the river trial and 2‰ of the distance travelled (CEP) in the sea trials.

## Figures and Tables

**Table 1.** Inertial sensors specifications.

**Bias**		**Scale Factor**		**Rate**
	
**Gyro**	**Acc**	**Gyro**	**Acc**	
0.03 deg/h	50 μg	10 PPM	50 PPM	200 Hz

**Table 2.** Primary navigation aiding sensors.

**Variable**	**Sensors**	**Precision**	**Rate**
Position	NovAtel DGPS	1 m	1 Hz
Velocity	HEU DVL	± 0.5% ± 0.5 cm/s	>=1 Hz

**Table 3.** Calibrated parameter estimates in the river trial.

	**N1**			**N2**			**N3**			**N4**		

	**1** + **k**	***α***	***β***	**1** + **k**	***α***	***β***	**1** + **k**	***α***	***β***	**1** + **k**	***α***	***β***
Initial estimates	0.9935	0.075	0.256	0.9988	0.287	0.363	0.9954	0.265	0.267	0.9958	0.423	0.207
iterative estimates	0.9944	0.123	0.248	0.9936	0.134	0.365	0.9972	0.271	0.244	0.9960	0.390	0.214

**Table 4.** Validation of Calibrated parameter estimates.

	**N1**		**N2**		**N3**		**N4**	

**D(m)**	**Position Error(m)**	**Accuracy (‰D)**	**Position Error(m)**	**Accuracy (‰D)**	**Position Error(m)**	**Accuracy (‰D)**	**Position Error(m)**	**Accuracy (‰D)**
7,140	3.0	0.40	4.0	0.56	5.0	0.70	3.0	0.40

**Table 5.** On-line experimental results in the river trial.

	**N1**		**N2**		**N3**		**N4**	

**D(km)**	**Position Error (m)**	**Accuracy (‰D)**	**Position Error(m)**	**Accuracy (‰D)**	**Position Error(m)**	**Accuracy (‰D)**	**Position Error(m)**	**Accuracy (‰D)**
20	34.7	1.73	34.9	1.74	35.9	1.79	36.2	1.81
40	31.7	0.79	58.3	1.45	41.4	1.03	52.7	1.32
60	18.2	0.3	66.8	1.11	30.8	0.5	54.9	0.91
80	37.3	0.46	70.8	0.88	46.8	0.58	62.6	0.78
100	42.3	0.47	88.6	0.98	53.1	0.58	80.7	0.89

**Table 6.** On-line experimental results in Experiment 1.

**Distance (m)**	**Error (m)**	**Accuracy (‰D)**
17,614	31.0	1.76

**Table 7.** On-line experimental results in Experiment 2.

**Distance (m)**	**Error (m)**	**Accuracy (‰D)**
31,467	53.3	1.7
29,772	90.4	3.0
31,102	68.5	2.2
31,870	56.0	1.7

**Table 8.** On-line experimental results in Experiment 3.

**Distance (m)**	**Error (m)**	**Accuracy (‰D)**
30,836	90.3	2.9
60,341	27.0	0.5
